# Usefulness of Percutaneous Drainage in Determining the Causative Microorganism in Patients with Spondylodiscitis: A Retrospective Cross-Sectional Study

**DOI:** 10.5334/jbsr.3127

**Published:** 2023-07-10

**Authors:** Shoichi Ikenaga, Daisuke Yunaiyama, Mika Yasutomi, Itaru Nakamura, Mitsuru Okubo, Toru Saguchi, Motoki Nakai, Kazuhiro Saito

**Affiliations:** 1 Department of Radiology, Tokyo Medical University, Tokyo Medical University Hospital, 6-7-1 Nishishinjuku, Shinjuku-ku, Tokyo, 160-0023, Japan; 2 Department of Infection Prevention and Control, Tokyo Medical University Hospital, 6-7-1 Nishishinjuku, Shinjuku-ku, Tokyo, 160-0023, Japan

**Keywords:** spondylodiscitis, drainage, infection

## Abstract

**Purpose:**

To determine the usefulness of CT-guided percutaneous drainage for the causative microorganism detection in patients with spondylodiscitis.

**Materials and Methods:**

Data of patients who underwent CT-guided percutaneous drainage for spondylodiscitis from January 2014 to April 2022 were extracted from the radiological database of our hospital and investigated. The administration rate of antibiotics prior to blood culture and CT-guided percutaneous drainage (CTPD) were analyzed. The detection rate of microorganisms via blood culture and CT-guided percutaneous drainage were compared using the Mann–Whitney’s U test with the SPSS software.

**Results:**

In this study, a total of 30 (20 male and 10 female) patients were analyzed. A total of 13 patients (43%) were administered antibiotics prior to blood culture. Of them, microorganisms were detected via blood culture in only one patient (7%). A total of 25 patients (83%) were administered antibiotics prior to CTPD. Of them, the causative microorganisms in 19 patients (76%) were detected. Overall, the causative microorganism could be detected in 24 out of 26 patients (92%) via CT-guided percutaneous drainage. There was a statistical significance in the detection rate of microorganisms between blood culture and CTPD (P = 0.004) in favor of CTPD.

**Conclusion:**

CT-guided percutaneous drainage showed a high positive rate of microorganism detection in patients with spondylodiscitis regardless of antibiotic administration prior to the procedure. CT-guided percutaneous drainage can be a solution for the detection of the causative microorganism in spondylodiscitis patients who received antibiotics before obtaining any culture.

## Introduction

Spondylodiscitis is an infectious inflammatory disease occurring in the vertebral disc with the incidence of 2.4 per 10,000 people [1,2,3]. The causative microorganism of spondylodiscitis may enter into the vertebral disc via blood stream infection due to iatrogenic causes or wounds from the surrounding tissue of the vertebral body or distant area [4,5]. Vertebral disc inflammation also easily extends to adjacent vertebral bodies thus resulting in osteomyelitis, which causes abscess formation in the surrounding tissue or in the epidural space with a mortality rate of 6% without medical intervention [1,2,4].

Microorganism detection is performed via blood culture, which is one of the most important techniques for infection control or any other specimen cultures [6]. Hopkinson et al. reported that the mean antibiotic administration period was shortened from 142–77 days by identifying the causative microorganism [7]. This demonstrates that the identification of the causative microorganism is the most appropriate therapeutic intervention for patients with spondylodiscitis. However, the positive rate of blood culture in patients with clinically diagnosed spondylodiscitis was 40%–60% [6]; moreover, the positive rate decreases in the range between 20.6%–27.7% in patients receiving antibiotics administration before taking blood culture [8]. Therefore, prompt diagnosis by blood culture before antibiotics administration is essential to manage patients with spondylodiscitis.

CT-guided percutaneous drainage (CTPD) is a technique that involves tube insertion into the vertebral disc space as well as specimen sampling and continuous drainage from the infected site simultaneously [9]. The rate of microorganism detection via insertion of the drainage tube into the infected vertebral disc space was reported to be 76.2%–83.3% [9,10]. We hypothesized that CTPD could reveal the causative microorganism in patients who received antibiotics before blood culture as the vertebral disc is less vascularized and the immune reaction or antibiotics are not effective [5]. This study aimed to investigate the usefulness of CTPD to the inflammatory vertebral disc space or adjacent tissue focusing on the detection rate of the causative microorganism in patients with or without antibiotics administration prior to the intervention.

## Materials and Methods

### Ethics

This retrospective cross-sectional study was approved by the ethical board of our institution, which waived the requirement to obtain informed consent.

### Materials

**Figure 1:**
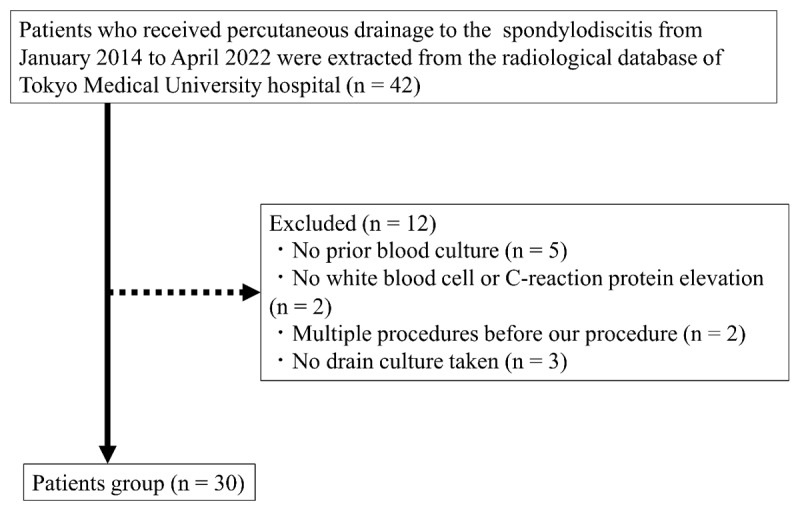
Summary of patient selection.

Patient selection was performed as presented in Figure 1. Patients who underwent CTPD for spondylodiscitis from January 2014 to April 2022 were extracted from the radiological database of our hospital. CTPD is performed in patients with spondylodiscitis in our hospital to determine the causative bacteria and decrease the amount of abscess formation. The exclusion criteria were as follows: (1) no blood culture prior to the CTPD, (2) no white blood cell (WBC) or C-reactive protein (CRP) elevation observed in the blood biochemical test, (3) multiple procedures in a patient before our procedure and (4) no culture by a puncture taken.

### CT-guided percutaneous drainage

The patient should be set in the prone position on the bed of the interventional radiology system (INFX-8000C, Canon Medical Systems Inc., Tokyo, Japan). Consecutively CT scan was obtained, then the target lesion was revealed. Sequentially, CT fluoroscopy was used to perform a puncture to the target lesion. The protocol was as follows; 80 or 120 kV, 40 mAs, 2–4 mm slice thickness.

**Figure 2:**
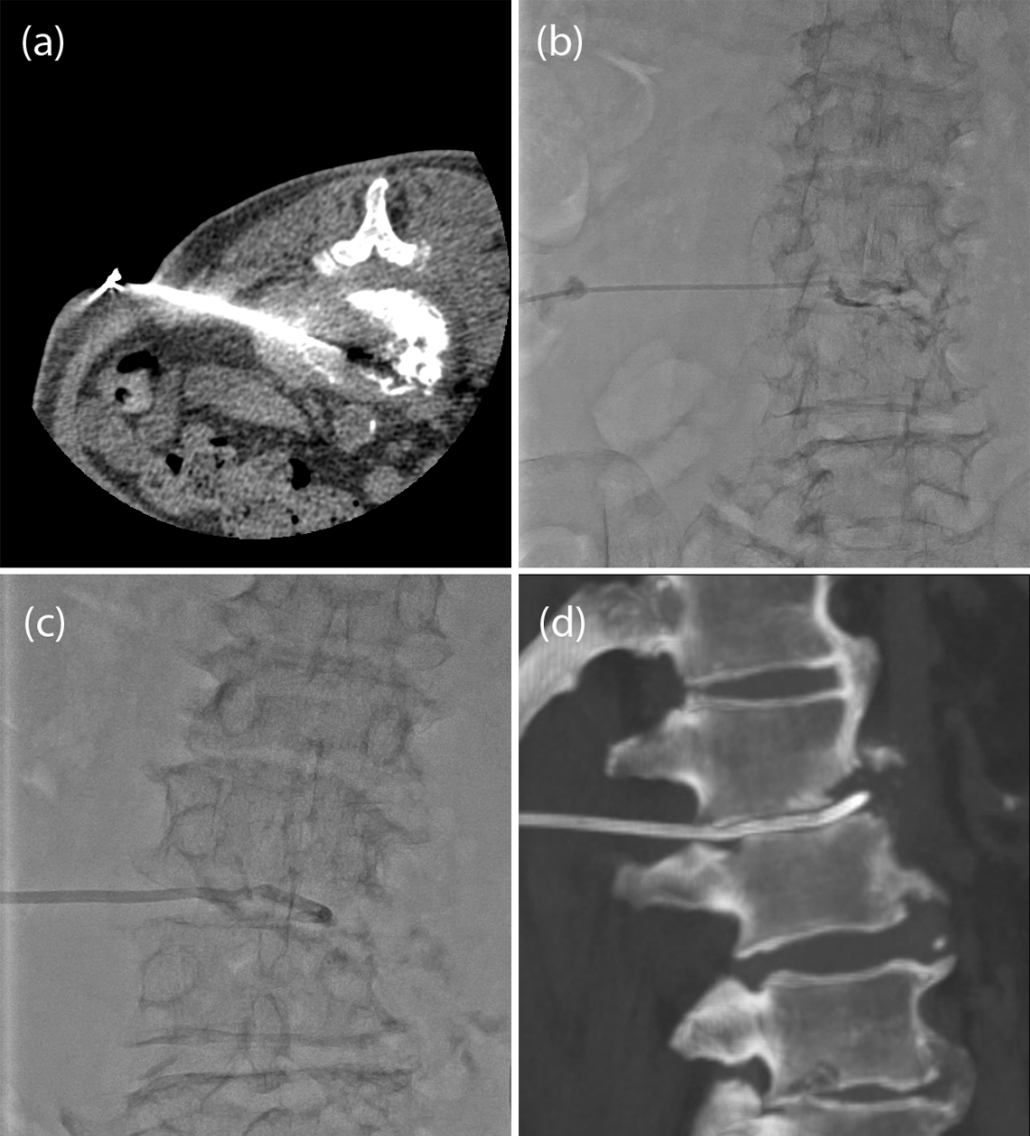
Patients who underwent intradiscal and intrapsoas muscle drainage tube placement via CT-guided percutaneous insertion. **(a)** CT image showing the procedure for creating puncture to the disc space using a 18G Surflo needle guided by CT in the prone position under local anesthesia with procaine. **(b)** Fluoroscopy image demonstrating the injection of contrast-enhanced material through the outer sheath of the Surflo needle and then pulling it out. **(c)** Fluoroscopy image demonstrating a drainage tube being inserted into the discal space. A stiff-type guidewire (Amplatz ultrastiff guidewire, COOK medical Japan, Tokyo, Japan) was used. **(d)** A reconstructed CT image after the procedure showing that the drainage tube was located in the discal space.

A representative image obtained during a CTPD procedure is presented in Figure 2. The parameters in this study investigated were (1) place of drainage tube placement, (2) type or shape of the drainage tube, (3) success rate of the drainage tube placement, and (4) rate of adverse events.

Patient characteristics such as age, sex, medical history, the presence or absence of antibiotic administration prior to blood culture and CTPD, the microorganism incubated by cultured from the drained specimen, the period of drainage tube placement, and the duration time of antibiotic administration were obtained from the medical records. Blood and biochemical data, such as white blood cell (WBC), C-reactive protein (CRP), and creatinine (Cre) values prior to CTPD were also obtained.

The incidence of an adverse event due to CTPD was analyzed, which was observed during and after procedure.

### Statistical analysis

The relationship between the microorganism detection rate and antibiotic administration was investigated and analyzed using the X^2^ test. The patient characteristics for the positive or negative detection of microorganisms via blood culture and CTPD were compared using the Mann–Whitney’s U test. All the statistical analysis was performed with the SPSS software (ver. 28, SPSS Inc., Chicago, IL, USA).

## Results

In this study, a total of 30 patients (20 male and 10 female) with spondylodiscitis were analyzed. Their median age was 73 years old. The major medical history was diabetes mellitus (13 out of 30), followed by chronic renal failure (8 out of 30). The median Cre value was 0.82 mg/mL. In the 12 patients who had a positive blood culture, the median WBC was 10600/μL (range, 6000–22500/μL); CRP, 10.5 mg/mL (range, 0.5–25.7 mg/mL); and Cre, 0.81 mg/mL (range, 0.26–5.69 mg/mL). In the 18 patients who were negative for blood culture, the median WBC was 7500/μL (range, 3100–13300/μL); CRP, 6.2 mg/mL (range, 0.77–25.7 mg/mL); and Cre, 0.88 mg/mL (range, 0.26–5.69 mg/mL).

In 24 patients who had a positive specimen obtained during CTPD, the median WBC was 8250/μL (range, 3600–22500/μL); CRP, 6.60 mg/mL (range, 0.77–25.7 mg/mL); and Cre, 0.92 mg/mL (range, 0.38–3.88 mg/mL). In the six cases who had a negative specimen obtained during CTPD, the median WBC was 6900/μL (range, 3100–13300/μL); CRP, 2.31 mg/mL (range, 0.50–14.7 mg/mL); and Cre, 0.49 mg/mL (range, 0.26–5.69 mg/mL). The patient characteristics in groups of positive or negative specimens after CTPD were summarized in Table 1.

**Table 1:** Summary of patient characteristics and clinical manifestation according to the results of CT-guided percutaneous drainage.


CTPD	BC POSITIVE: NEGATIVE	SEX (M: F)	MEDIAN NUMBER AND RANGE				UNDERLYING DISEASE

			AGE	CRP (MG/ML)	WBC (/μL)	CRE (MG/ML)	

Positive	10:14	19:5	73(21–91)	6.60(0.77–25.7)	8250(3600–22500)	0.92(0.38–3.88)	LC (2), OMI (2), CRT-D, CKD (7), renal cancer, HT, DM (11), HT (6), CI, esophageal cancer, spinal canal stenosis, uterine cancer, HCV (2), RA, tuberculosis, bladder cancer, MCTD, atopic dermatitis (2), HIV, hemophilia, angina pectoris, HL (2), CHF, Af (2), AKI, CLD, psoriasis

Negative	2:4	1:5	70.5(62–73)	2.31(0.05–14.7)	6900(3100–13300)	0.49(0.26–5.69)	DM (2), CKD, HT, osteoporosis, uterine myoma, glaucoma, knee osteoarthritis, lumber hernia


CTPD: CT-guided percutaneous drainage, BC: blood culture, M: male, D: female, LC: liver sclerosis, OMI: old myocardial infarction, CRT-D: cardiac resynchronization therapy-defibrillator, CKD: chronic kidney disease, HD: hemodialysis, DM: diabetes mellitus, CI: cerebral infarction, HCV: hepatitis type C virus, HT: hypertension, RA: rheumatoid arthritis, MCTD: mixed connective tissue disease, HIV: human immunodeficiency virus, HL: hyperlipidemia, CHF: chronic heart failure, AKI: acute kidney injury, Af: atrial fibrillation, CLD: chronic liver disease.

The success rate of CTPD was 100%. The drainage tube was placed in the disc (71.3%), the psoas muscle (50.3%), or both (20.0%). The type and shape of the placed catheter are summarized in Table 2.

**Table 2:** Summary of the drainage place, shape and size of the drainage tube, and tube insertion duration in each patient.


DRAINAGE PLACE	NUMBER OF PATIENTS	DRAINAGE TUBE SHAPE	SIZE (FR.)	TUBE INSERTION DURATION (DAYS)

Disk	16	Straight: 4	6–8*	7–11

		Pigtail: 12	8–10	4–35

Psoas	8	Straight	6.3–14	21–23

		Pigtail	8–14	5–38

Disk and psoas	6	Pigtail	8–10	7–26


*: Includes outer sheath of a 18G Surflo needle.

Only one patient had an adverse event. In this patient, puncture to the disc space was successful; however, the patient was supposed to have suffered from septic shock after injection of contrast-enhanced material to the disc space as the patient showed shivering, decreased blood pressure, and elevated body temperature. The outer sheath of the puncture needle in this patient was placed in the disc space to perform continuous drainage.

The median period of the tube placement in all patients was 11.5 days.

### Microorganism detection rate between blood culture and CT-guided percutaneous drainage

Blood culture could reveal the causative microorganism in 12 of 30 patients (40%). A total of 13 of 30 patients (43%) were administered antibiotics prior to blood culture. Of them, microorganisms were detected via blood culture in only one patient (7%).

CTPD could reveal the causative microorganism in 24 of 30 patients (80%). A total of 25 patients (83%) were administered antibiotics prior to CTPD. Of them, the causative microorganisms was revealed in 19 patients (76%) by CTPD.

Among 18 patients whose blood cultures were negative, the causative microorganism was detected by CTPD in 14 patients (78%). Only two of 30 patients (7%) showed a positive blood culture and had a negative specimen after CTPD.

As mentioned previously, a total of 13 patients were administered antibiotics prior to blood culture. Of them, microorganisms were detected via blood culture in only one patient (7%). In contrast, 17 patients were not administered antibiotics prior to blood culture, and the microorganism in 12 patients (70%) was detected using CTPD.

The causative microorganisms in 19 of the 25 patients (76%) who received antibiotics prior to CTPD were detected from the specimen obtained via CTPD.

### Species of the detected microorganism and the clinical course

The species of the detected microorganisms via CTPD are summarized in Table 3. Microorganisms not detected by CT-guided percutaneous drainage are summarized in Table 4. In two patients, the causative microorganisms were not detected by CTPD, but by blood culture. Neither blood culture nor CTPD revealed the causative microorganisms in four patients. Overall, the causative microorganisms could be detected in 26 out of 30 (87%) patients via the sequence of blood culture and CTPD.

**Table 3:** Summary of microorganisms detected via CT-guided percutaneous drainage.


	NUMBER OF PATIENTS	PRE-CTPD ANTIBIOTIC ADMINISTRATION (+ : –)

***Escherichia coli***	6	5:1

**MSSA**	4	3:1

**MRSA**	3	3:0

***Klebsiella pneumoniae***	2	2:0

***Enterococcus faecalis***	2	1:1

***Tuberculosis***	1	0:1

***Mycobacterium intracellulare***	1	0:1

***Candida albicans***	1	0:1

***Streptococcus anginosus***	1	0:1

***Citrobacter koseri***	1	1:0

***Staphylococcus epidermis***	1	1:0

***Streptococcus species***	1	1:0


MSSA: methicillin-resistant staphylococcus aureus, MRSA: methicillin-resistant staphylococcus aureus.

**Table 4:** Summary of microorganisms not detected by CT-guided percutaneous drainage.


	NUMBER OF PATIENTS	PRE-CTPD ANTIBIOTIC ADMINISTRATION (+ : –)

**Unknown**	4	4:0

**MRSE**	1	1:0

***S. intermedius***	1	1:0


CTPD: CT-guided percutaneous drainage, MRSE: methicillin-resistant staphylococcus epidermis, S: Streptococcus.

### Statistical analysis results

Significant differences were observed in the value of WBC (*P* = 0.039) between patients’ group with positive or negative blood culture results. Significant differences were observed in the value of CRP (*P* = 0.043) between patients’ group with CTPD positive or negative. There was a statistically significant difference between the administration of antibiotics prior to blood culture and the microorganism detection rate (*P* = 0.002). There was a statistically significant difference in the microorganism detection rate in patients who received antibiotics prior to CTPD procedure compared to those who did not (*P* < 0.001). There was a statistical significance in the detection rate of microorganisms between blood culture and CTPD (P = 0.004) in favor of CTPD.

## Discussion

Our detection rate of the causative microorganism after CTPD was higher than in a the previously reported systematic review of image guided biopsy in infectious discitis [11]. This is because more pus can be obtained when using a thick drainage tube during a CT guided procedure than with a fluoroscopic needle biopsy; therefore, CTPD is a better option than fluoroscopically guided biopsy for the detecting of the causative microorganism and for performing drainage as it is capable of performing abscess drainage in the adjacent soft tissues [9]. Furthermore, percutaneous CT-guided drainage is lined up as a second option together with endoscopic drainage and surgical drainage when fluoroscopically guided needle biopsy was negative according to the Infectious Diseases Society of America [12]. Endoscopic drainage and surgical drainage require general anesthesia which makes CTPD a better second option as it is performed under local anesthesia and is less invasive.

A systematic review reported that the positive culture rate of pyogenic discitis by fluoroscopically guided biopsies and open surgical biopsy were 48% and 76%, respectively [11]. Our positive microorganism detection rate of 80% after CTPD in patients with purulent discitis was comparable with surgical biopsy, suggesting that CTPD would be a good option for microorganism detection before surgery is attempted, as CTPD is less invasive.

In our study, the values of WBC and CRP were significantly correlated with the microorganism detection rate via blood culture and CTPD, respectively. To the best of our knowledge, there is no previous report showing that clinical data have an influence on the causative microorganism detection rate on CTPD. High CRP values indicate severe inflammatory reaction in the region of spondylodiscitis, which may correlate with a dense concentration, causative microorganisms at the infection site. Contrarily, Seigel et al. reported that the WBC count was inadequate in the prediction of bacteremia in patients with suspected infection [13]. In our opinion, the possible reason for this may be that many patients whose blood culture was negative in their study received antibiotics before blood culture due to which their WBC (and CRP) values may have decreased. Therefore, additional studying with greater number of patients with spondylodiscitis without antibiotics administration before CTPD is required to further investigate the correlation between clinical data (such as WBC and CRP values) and the prediction of culture positivity after CTPD.

This study has several limitations. First, this is a retrospective study; thus, selection bias might be present. Second, the materials in this study might include non-infectious spondylodiscitis as this diagnosis was suggested by clinical information, such as CT and MRI findings. The patients in our study showed hyper intensity of vertebral body and disc on T2-weighted MRI images with edematous adjacent soft tissue. This not necessarily reflects infection and might also be due to degenerative disease. Third, a negative culture after CTPD cannot exclude an infectious spondylodiscitis because of possible procedure failure. Fourth, only a small number of patients were included in this study.

In conclusion, CTPD at our institution had a high positive microorganism detection rate in patients with spondylodiscitis regardless of the status of antibiotic administration prior to this procedure.
